# Driver’s Licensure and Driving Outcomes Among Youths With Mood Disorders

**DOI:** 10.1001/jamanetworkopen.2024.5543

**Published:** 2024-04-08

**Authors:** Christopher E. Gaw, Kristina B. Metzger, Melissa R. Pfeiffer, Benjamin E. Yerys, Rhonda C. Boyd, Daniel J. Corwin, Allison E. Curry

**Affiliations:** 1 Division of Emergency Medicine, Nationwide Children’s Hospital, Columbus, Ohio; 2 Center for Injury Research and Prevention, Children’s Hospital of Philadelphia, Philadelphia, Pennsylvania; 3 Center for Autism Research, Children’s Hospital of Philadelphia, Philadelphia, Pennsylvania; 4 Department of Child and Adolescent Psychiatry and Behavioral Sciences, Children’s Hospital of Philadelphia, Philadelphia, Pennsylvania; 5 Department of Psychiatry, Perelman School of Medicine at the University of Pennsylvania, Philadelphia, Pennsylvania; 6 Division of Emergency Medicine, Children’s Hospital of Philadelphia, Philadelphia, Pennsylvania

## Abstract

**Importance:**

Mood disorders are prevalent among adolescents and young adults, and their onset often coincides with driving eligibility. The understanding of how mood disorders are associated with youth driving outcomes is limited.

**Objective:**

To examine the association between the presence of a mood disorder and rates of licensing, crashes, violations, and suspensions among adolescents and young adults.

**Design, Setting, and Participants:**

This cohort study was conducted among New Jersey residents who were born 1987 to 2000, age eligible to acquire a driver’s license from 2004 to 2017, and patients of the Children’s Hospital of Philadelphia network within 2 years of licensure eligibility at age 17 years. The presence of a current (ie, ≤2 years of driving eligibility) mood disorder was identified using *International Classification of Diseases, Ninth Revision, Clinical Modification *(*ICD-9-CM*) or *International Statistical Classification of Diseases, Tenth Revision, Clinical Modification *(*ICD-10-CM*) codes. Rates of licensure and driving outcomes among youths who were licensed were compared among 1879 youths with and 84 294 youths without a current mood disorder from 2004 to 2017. Data were analyzed from June 2022 to July 2023.

**Main Outcomes and Measures:**

Acquisition of a driver’s license and first involvement as a driver in a police-reported crash and rates of other adverse driving outcomes were assessed. Survival analysis was used to estimate adjusted hazard ratios (aHRs) for licensing and driving outcomes. Adjusted rate ratios (aRRs) were estimated for driving outcomes 12 and 48 months after licensure.

**Results:**

Among 86 173 youths (median [IQR] age at the end of the study, 22.8 [19.7-26.5] years; 42 894 female [49.8%]), there were 1879 youths with and 84 294 youths without a mood disorder. A greater proportion of youths with mood disorders were female (1226 female [65.2%]) compared with those without mood disorders (41 668 female [49.4%]). At 48 months after licensure eligibility, 75.5% (95% CI, 73.3%-77.7%) and 83.8% (95% CI, 83.5%-84.1%) of youths with and without mood disorders, respectively, had acquired a license. Youths with mood disorders were 30% less likely to acquire a license than those without a mood disorder (aHR, 0.70 [95% CI, 0.66-0.74]). Licensed youths with mood disorders had higher overall crash rates than those without mood disorders over the first 48 months of driving (137.8 vs 104.8 crashes per 10 000 driver-months; aRR, 1.19 [95% CI, 1.08-1.31]); licensed youths with mood disorders also had higher rates of moving violations (aRR, 1.25 [95% CI, 1.13-1.38]) and license suspensions (aRR, 1.95 [95% CI, 1.53-2.49]).

**Conclusions and Relevance:**

This study found that youths with mood disorders were less likely to be licensed and had higher rates of adverse driving outcomes than youths without mood disorders. These findings suggest that opportunities may exist to enhance driving mobility in this population and elucidate the mechanisms by which mood disorders are associated with crash risk.

## Key Points

**Question:**

What is the association of mood disorders with license acquisition and first-time crash and other adverse driving outcomes among adolescents and young adults?

**Findings:**

In this cohort study of 1879 youths with and 84 294 youths without a mood disorder, the rate of licensure was 30% lower among those with mood disorders. Youths with mood disorders had higher adjusted rates of moving violations and license suspensions compared with peers without mood disorders.

**Meaning:**

These findings suggest that opportunities may exist to improve driving autonomy among youths with mood disorders while concurrently ensuring safe mobility.

## Teaser

This cohort study investigates the association of mood disorders with driver’s license acquisition and adverse driving outcomes among adolescents and young adults in New Jersey.

## Introduction

Mood disorders are common mental health disorders with consequences for physical well-being and psychosocial functioning.^1,2,3,4^ Among adolescents and young adults, rates of health care encounters for mood disorders have been increasing,^5,6^ and the 12-month prevalence of both mood disorders in this population may be as high as 11% in the US.^4,6^ Notably, the incidence of mood disorders increases in the postpubertal period,^1,3,4^ which coincides with the time when many adolescents begin to consider obtaining a license to drive.^7,8^

Driving requires multiple neurocognitive skills, including executive functioning, sensory perception, and attention^9,10,11^; these skills are often impaired in individuals with mood disorders.^12,13,14^ Adult drivers with depression demonstrated slower steering wheel reaction time and deceleration rates in driving simulators compared with counterparts without depression.^15,16^ Several studies of adult drivers have reported positive associations between mood disorders and traffic violations,^17^ license loss,^18^ and motor vehicle crashes.^19,20,21,22^ Hill et al^23^ examined a subset of studies that used an epidemiological design; using a pooled analysis, the authors estimated that the odds of crash involvement among adult drivers with depression was 90% higher than among drivers without depression.

Adolescent and young adult drivers demonstrate increased, developmentally related crash risks compared with older adults,^9,10,24,25^ and those with mood disorders may be at higher risk for motor vehicle crashes. Despite these concerns, the literature examining this issue among adolescents and young adults is limited. Although a prior naturalistic study^26,27^ included adolescents, adjusted analyses specifically on adolescents were not performed, and the study relied on self-reports of depressive symptoms. Additionally, licensure is an important life milestone for many adolescents; acquiring a license to drive can reduce social isolation and foster independent mobility, which can have psychosocial benefits. To our knowledge, longitudinal studies of licensure acquisition and crash or other adverse driving outcomes of adolescents and young adults with diagnosed mood disorders have not been undertaken.

We conducted a cohort study to examine driving outcomes among adolescents and young adults with and without mood disorders. Specifically, we compared rates of driving licensure and, over the first 4 years of licensure among licensed youths, driving outcomes, including rates of police-reported crashes, moving violations, and license suspensions. To do this, we identified a large study cohort from the New Jersey Safety and Health Outcomes (NJ-SHO) Data Warehouse, which integrates electronic health records (EHRs) of patients at the Children’s Hospital of Philadelphia (CHOP) and New Jersey statewide traffic data.^28^ Results of this foundational study may inform guidance for families with youths who have mood disorders and identify opportunities for future research.

## Methods

The CHOP Institutional Review Board approved this cohort study and granted a waiver of Health Insurance Portability and Accountability Act authorization and informed consent. Given the retrospective nature of the study, approaching all individuals whose data were contained in the final database was infeasible. We adhered to the best practices outlined in the Strengthening the Reporting of Observational Studies in Epidemiology (STROBE) reporting guideline.

### Study Design and Sample

We identified youths for this study from the CHOP pediatric health care network, which serves a socioeconomically diverse patient population at more than 50 sites throughout southeastern Pennsylvania and southern New Jersey. CHOP clinicians manage all aspects of clinical care using a unified, linked EHR system (EpicCare; Epic Systems, Inc). We queried the CHOP EHR system to select youths who were born from 1987 to 2000 and were patients at a CHOP health care network site as a New Jersey resident within 4 years prior to becoming eligible for their intermediate driver’s license at age 17 years (ie, ages 13-16 years).

### Exclusion Criteria and Mood Disorder Classification

**Figure 1.**
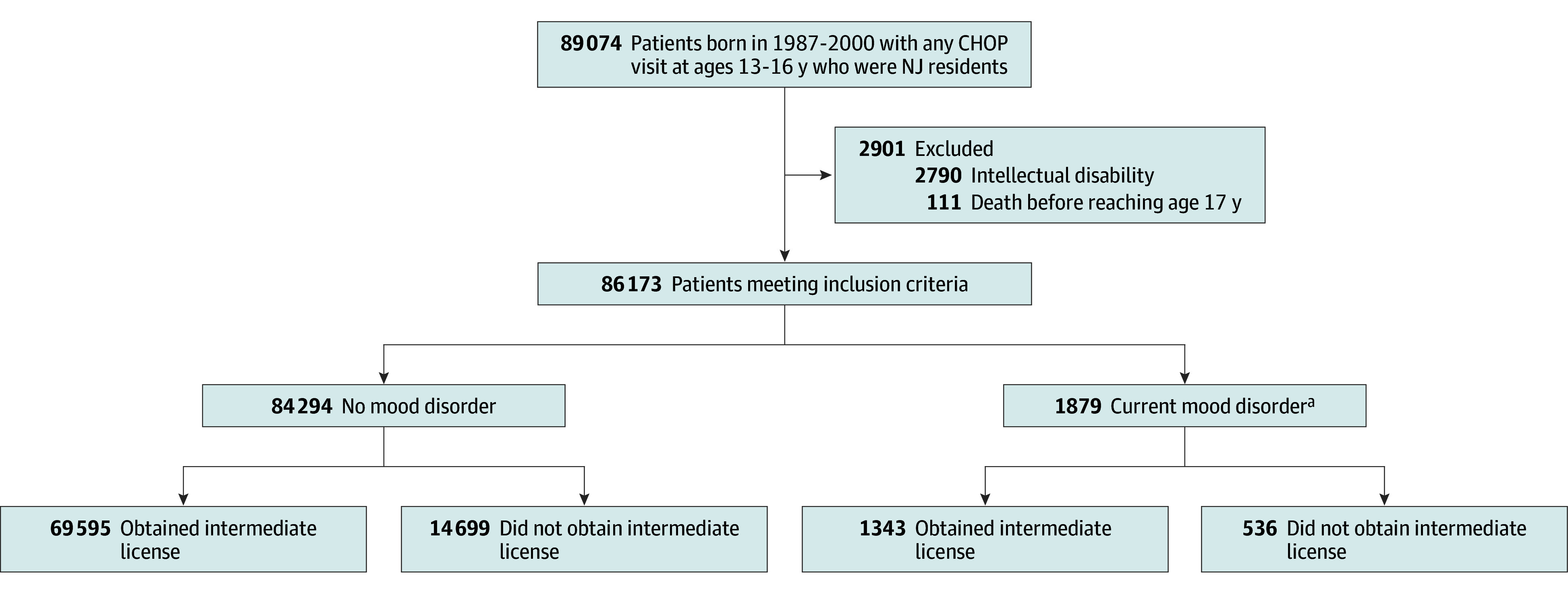
Study Flowchart ^a^Current mood disorder was defined as a documented *International Classification of Diseases, Ninth Revision, Clinical Modification *(*ICD-9-CM*) or *International Statistical Classification of Diseases, Tenth Revision, Clinical Modification *(*ICD-10-CM*) diagnosis or problem list code at a Children’s Hospital of Philadelphia (CHOP) visit at ages 15 to 16 years.

Of 89 074 youths selected, we excluded 2790 individuals (3.1%) who had a diagnosed intellectual disability. An intellectual disability diagnosis was defined as the presence of a corresponding *International Classification of Diseases, Ninth Revision, Clinical Modification *(*ICD-9-CM*) or *International Statistical Classification of Diseases, Tenth Revision, Clinical Modification *(*ICD-10-CM*) diagnostic code at a CHOP EHR visit or on the patient list of known medical conditions (ie, problem list). We excluded an additional 111 youths (0.1%) who died prior to their seventeenth birthday. Thus, the underlying study cohort included 86 173 youths (Figure 1).

We then identified 1879 youths (2.2%) within our cohort with current mood disorders (Figure 1). A current mood disorder was defined as a documented *ICD-9-CM* or *ICD-10-CM* diagnosis or problem list code at a CHOP visit within 2 years of license eligibility (ie, at ages 15-16 years). We used this age range to minimize the capture of patients with mood disorders that had resolved or were in remission by age 17 years. *ICD-9-CM* and *ICD-10-CM* codes were bridged using General Equivalence Mappings provided by the US Centers for Medicare & Medicaid Services (see the complete list in eTable 1 in Supplement 1).^29^

### Data Linkage

We obtained traffic safety data for 2004 to 2017 from the New Jersey Motor Vehicle Commission Licensing Database and New Jersey Department of Transportation Crash Database. Collectively, these databases contain detailed information on all licensed drivers, license suspension and traffic violation events, and police-reported crashes that occur within the state. We linked CHOP EHR data to New Jersey traffic safety data during the development of the NJ-SHO data warehouse via a probabilistic linkage using common data elements, which included full name, exact date of birth, and residential address. Full details regarding the construction and evaluation of the NJ-SHO data warehouse are described elsewhere.^28^

### Study Outcomes

Time in months to acquisition of a New Jersey driver’s license was assessed for each youth in the cohort. The minimum licensure age in New Jersey is 17 years, at which time adolescents are eligible for an initial intermediate (eg, with passenger and night restrictions) driver’s license.^30^ The primary driving outcome was time from licensure to first involvement as a driver in a New Jersey police-reported crash in months. A crash is reportable if an injury or more than $500 in property damage occurred.^31^ Follow-up time concluded with death, license expiration, or end of the study period, whichever occurred first. We also assessed the mean monthly rate of all police-reported crashes over the first 12 and 48 months after licensure for our cohort. In addition, we calculated mean monthly rates for the first 12 and 48 months of 4 secondary driving outcomes: at-fault crashes, injury crashes, night crashes, and peer-passenger crashes. The numerator for each monthly crash rate was the number of days in the month with a police-reported crash among licensed drivers; the denominator, expressed as person-time, was calculated as the sum for all drivers of the proportion of the month that each driver had a valid license. For drivers who crashed on more than 1 day in a month, all days with crashes were included in the numerator. We defined at-fault crashes as those in which our cohort member had at least 1 crash-contributing driver action (eg, unsafe speed or improper lane change).^32^ Injury crashes included crashes where at least 1 individual had an injury of moderate or greater severity (as noted on the crash report). Nighttime crashes occurred from 9:01 pm through 4:59 am, which is designated as a period of higher risk for newly licensed youth drivers. Peer-passenger crashes involved crashes in which the driver was carrying only passengers aged 14 to 20 years, which has been associated with increased crash risk for this group.^33^ Using similar methods, we calculated moving violation citations (eg, speeding or careless driving), graduated driver licensing (GDL) violations (for intermediate drivers aged <21 years), and annual rates of license suspension, calculated as the number of days with a suspended license per year.

### Other Variables

The following demographic variables were obtained from the linked data: date of birth, sex, race and ethnicity, and insurance payor at last CHOP visit. Race and ethnicity were obtained from separate variables in the CHOP EHR. Ethnicity value options were a single selection: Hispanic or Latino, not Hispanic or Latino, refused, unknown, or null. Race value options were a multiple selection: American Indian or Alaska Native, Asian or Indian, Black or African American, Native Hawaiian or Other Pacific Islander, White, multiple races, other, refused, or null. If Hispanic or Latino was indicated, participants were classified as Hispanic regardless of race values indicated. All other participants who chose a single race category were classified as that racial group (eg, non-Hispanic White or non-Hispanic Black). Otherwise, participants who chose multiple race categories were classified as multiracial (eg, non-Hispanic multiracial). Age at licensure was calculated using date of birth and intermediate licensure date. We geocoded the residential address of each driver using crash reports as the primary source and licensing data as the secondary source to corresponding residential census tracts using ArcGIS software version 10.5.1 (Esri).^28^ Data from the 2010 Census Gazetteer Files and 2013 to 2017 American Community Survey 5-year estimates were used to construct quintiles of census tract–level population density and median household income, respectively.^34,35^ Individual race and ethnicity, which represent social constructs, are reported in this study to allow readers to assess generalizability of the study population and because they are related to health and societal inequities, which have historically led to differences in trajectories of mood disorder prevalence.^36,37^

### Statistical Analysis

We analyzed our study data from June 2022 to July 2023. We compared bivariate distributions of demographic characteristics among youths with and without mood disorders using the χ^2^ test for categorical variables and Wilcoxon rank-sum tests for continuous variables.

Driving outcomes were assessed with 2 complementary modes of analysis. We first used Kaplan-Meier survival curves to estimate cumulative probability of time to licensure from age of eligibility at 17 years and time to first crash from licensure; log-rank tests were used to compare differences. To assess the proportionality assumption, we considered the interaction of mood disorder status with follow-up time to licensure and to first crash; associations were not observed to vary temporally using linear or quadratic terms. We used multivariable Cox proportional hazard regression models to estimate adjusted hazard ratios (aHRs) with 95% CIs for licensure and driving outcomes. Adjusted models included potential covariates that were chosen a priori based on known or suspected mood disorder diagnosis and driving outcomes; these covariates included sex, year of birth, race and ethnicity, insurance payor, diagnosis of anxiety disorder, diagnosis of attention-deficit/hyperactivity disorder (ADHD), quintile of census tract–level median household income and population density, and age at licensure (for models of first crash only).

We then calculated monthly crash, violation, and suspension rates among licensed youths with and without mood disorders over the first 12 and 48 months of licensure. Estimates were restricted to drivers followed up to the specified postlicensure month. Adjusted rate ratios (aRRs) with associated 95% CIs were estimated using generalized estimated equation models with a log-link function to specify Poisson regression. Models accounted for repeated measures (eg, crashes, violations, or suspensions) within individual drivers using an independent working correlation matrix structure. Covariates for these models were chosen a priori and included sex, age at licensure, race and ethnicity, payor status, year of birth, diagnosis of anxiety disorder, diagnosis of ADHD, quintile of census tract–level median household income and population density, and linear and quadratic terms for month to control for temporal trends. We used 2-sided hypothesis testing to calculate *P* values. Analyses were conducted using SAS statistical software version 9.4 (SAS Institute). In this study, we did not conduct null hypothesis significance testing using a specific α level. Instead, point estimates with interval estimations are presented for adjusted models. This approach is in accordance with strong guidance from the American Statistical Association.^38^

## Results

### Demographic Characteristics

Among 86 173 youths (median [IQR] age, 15.0 [14.0-16.0] years at time of last CHOP visit and 22.8 [19.7-26.5] years at the end of the study period; 42 894 female [49.8%]; 8852 Black [10.3%], 8629 Hispanic [10.0%], and 61 549 White [71.4%]), there were 1879 youths with and 84 294 youths without a mood disorder. Demographic characteristics of youths with and without mood disorders were compared (Table; eTable 2 in Supplement 1). While half of the study population was female, almost two-thirds of youths diagnosed with mood disorders were female (1226 female [65.2%]) compared with 41 668 females (49.4%) among youths without mood disorders (*P* < .001). Youths with mood disorders were more likely to have a co-occurring anxiety disorder (573 youths [30.5%] vs 1194 youths [1.4%]) than youths without mood disorders. Most youths with mood disorders (1545 youths [82.2%]) had a depressive disorder only (ie, no bipolar disorder diagnosis).

**Table.** Demographic Characteristics by Mood Disorder Status^a^

Characteristic	Youths, No. (%)			*P* value
	Overall (N = 86 173)	Mood disorder (n = 1879)	No mood disorder (n = 84 294)	
Age, median (IQR), y				
At last visit before age 17 y	15.0 (14.0-16.0)	16.0 (16.0-16.0)	15.0 (14.0-16.0)	<.001
At end of study period	22.8 (19.7-26.5)	21.2 (18.8-24.8)	22.8 (19.8-26.5)	<.001
Sex				
Male	43 276 (50.2)	653 (34.8)	42 623 (50.6)	<.001
Female	42 894 (49.8)	1226 (65.2)	41 668 (49.4)	
Unknown	3 (<0.1)	0	3 (<0.1)	
Race and ethnicity				
Hispanic	8629 (10.0)	235 (12.5)	8394 (10.0)	<.001
Non-Hispanic Black	8852 (10.3)	126 (6.7)	8726 (10.4)	
Non-Hispanic White	61 549 (71.4)	1381 (73.5)	60 168 (71.4)	
Non-Hispanic other^b^	7113 (8.3)	137 (7.3)	6976 (8.3)	
Unknown	30 (<0.1)	0	30 (<0.1)	
Payor at last visit				
Private	74 408 (86.3)	1641 (87.3)	72 767 (86.3)	<.001
Medicaid	5943 (6.9)	179 (9.5)	5764 (6.8)	
Self-pay, not recorded, or not billed	5822 (6.8)	59 (3.2)	5763 (6.9)	
Median annual household income of residential census tract, $				
<48 506	6633 (7.7)	140 (7.5)	6493 (7.7)	.05
48 506-66 937	15 319 (17.8)	301 (16.0)	15 018 (17.8)	
66 938-84 921	22 324 (25.9)	525 (27.9)	21 799 (25.9)	
84 922-110 035	23 510 (27.3)	538 (28.6)	22 972 (27.3)	
≥110 036	18 377 (21.3)	375 (20.0)	18 002 (21.4)	
Unknown	10 (<0.1)	0	10 (<0.1)	
Neighborhood population density, population per square mile				
<1299	29 994 (34.8)	660 (35.1)	29 334 (34.8)	.18
1299-2919	27 439 (31.8)	623 (33.2)	26 816 (31.8)	
2920-5237	19 999 (23.2)	415 (22.1)	19 584 (23.2)	
5238-12 711	7537 (8.7)	165 (8.8)	7372 (8.7)	
≥12 712	1202 (1.4)	16 (0.9)	1186 (1.4)	
Unknown	2 (<0.1)	0	2 (<0.1)	
Co-occurring anxiety disorder at ages 15-16 y				
No	84 406 (97.9)	1306 (69.5)	83 100 (98.6)	<.001
Yes	1767 (2.1)	573 (30.5)	1194 (1.4)	
Co-occurring ADHD				
No	78 897 (91.6)	1367 (72.8)	77 530 (92.0)	<.001
Yes	7276 (8.4)	512 (27.2)	6764 (8.0)	

Abbreviation: ADHD, attention-deficit/hyperactivity disorder.

^a^ See eTable 2 in Supplement 1 for demographic characteristics of the study cohort by mood disorder status and sex.

^b^ Non-Hispanic other includes individuals who were categorized as either not Hispanic and 1 race (American Indian or Alaska Native, Asian or Indian, Native Hawaiian or Other Pacific Islander, or other) or not Hispanic and 2 or more of any race.

### Time to Licensure

**Figure 2.**
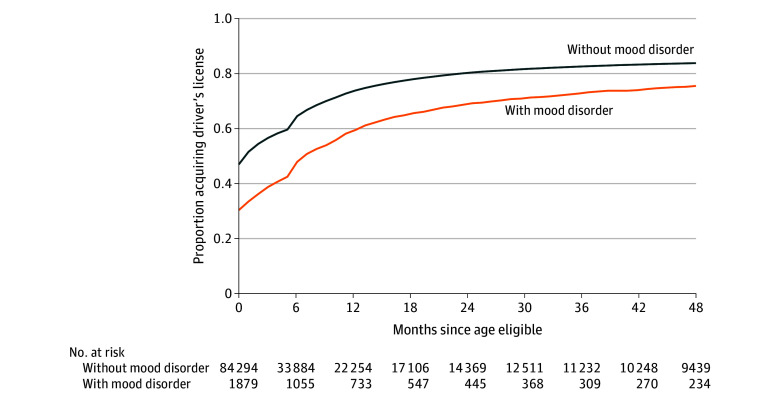
Inverse Kaplan-Meier Curves of Cumulative Probability of Licensure

In the first month after licensure eligibility, 570 youths with mood disorders (30.3% [95% CI, 28.3%-32.5%]) and 39 591 youths without mood disorders (47.0% [95% CI, 46.6%-47.3%]) had acquired a license to drive (Figure 2; eTable 3 and eTable 4 in Supplement 1). By 48 months after licensure eligibility, 1304 youths (75.5% [95% CI, 73.3%-77.7%]) and 68 409 youths (83.8% [95% CI, 83.5%-84.1%) with and without a mood disorder, respectively, had acquired a license. In adjusted Cox models, youths with mood disorders were 30% less likely to acquire a license compared with youths without a mood disorder (aHR, 0.70 [95% CI, 0.66-0.74]). Among licensed youths, those with and without mood disorders were of similar age at licensure (median [IQR] age, 17.2 [17.0-17.9] years and 17.0 [17.0-17.5] years).

### Time to First Crash

**Figure 3.**
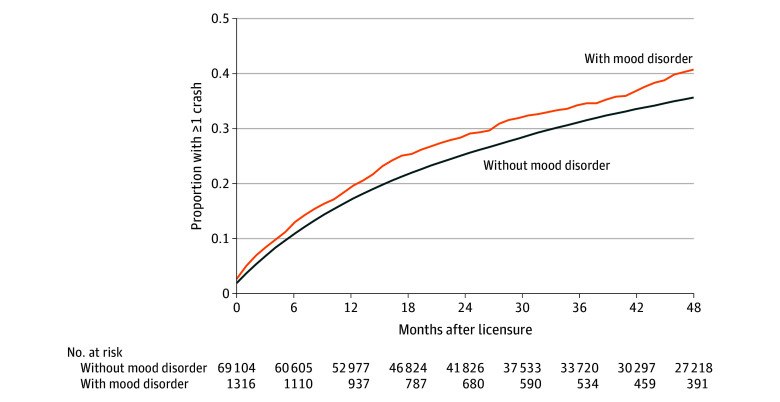
Inverse Kaplan-Meier Curves of the Cumulative Probability of First Crash Involvement Over Time

Overall, 1343 youths with mood disorders (71.5%) and 69 595 youths without mood disorders (82.6%) were licensed and thus followed up to determine driving outcomes. Among these individuals at 12 months after licensure, 227 individuals with mood disorders (18.4% [95% CI, 16.3%-20.6%) and 10 823 individuals without mood disorders (16.3% [95% CI, 16.1%-16.6%) were involved in their first crash (Figure 3; eTable 3 and eTable 4 in Supplement 1). By 48 months after licensure, 428 youths (40.7% [95% CI, 37.6%-43.9%) and 21 138 youths (35.6%; [95% CI, 35.2%-36.0%) with and without mood disorders, respectively, were involved in a crash. In adjusted Cox models, the risk of first crash among youths with and without mood disorders did not differ (aHR, 1.08 [95% CI, 0.99-1.19]).

### Rates of Crash Involvement and Circumstances

**Figure 4.**
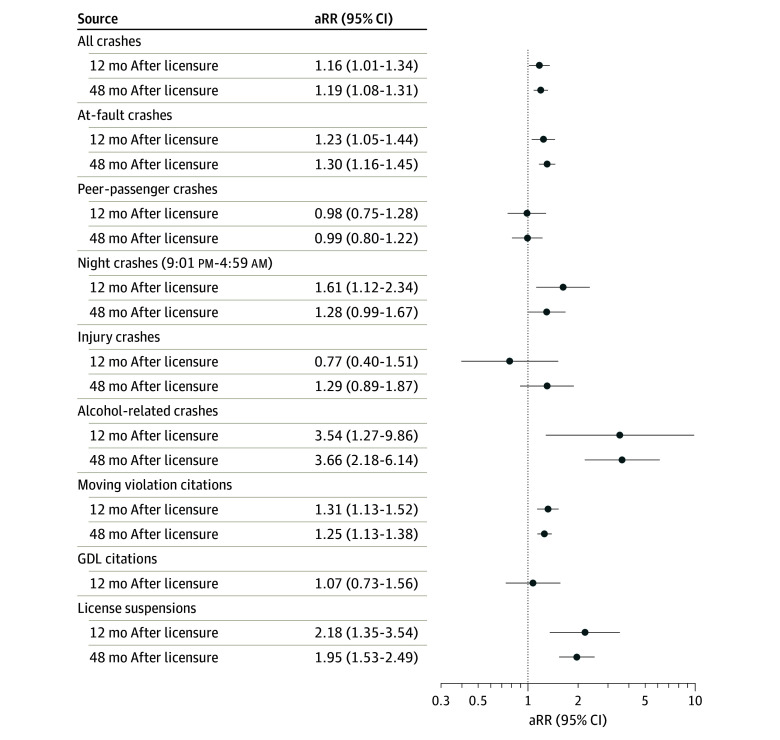
Adjusted Rate Ratios (aRRs) for Driving Outcomes Comparing Drivers With and Without Mood Disorders Dots indicate estimated aRRs; GDL, graduated driver licensing; lines, width of 95% CIs from Poisson regression models. See eTable 5 in Supplement 1 for crude RRs.

Drivers with mood disorders demonstrated higher overall crash rates than those without mood disorders at 12 months (185.3 vs 150.4 crashes per 10 000 driver-months) and 48 months (137.8 vs 104.8 crashes per 10 000 driver-months). Rates remained higher among individuals with mood disorders after adjusting for covariates at 12 months (aRR, 1.16 [95% CI, 1.01-1.34]) and 48 months (aRR, 1.19 [95% CI, 1.08-1.31]) (Figure 4; eTable 5 in Supplement 1). Rates of night crashes among drivers with mood disorders were increased at 12 months (aRR, 1.61 [95% CI, 1.12-2.34]) compared with those without mood disorders.

### Rates of Moving Violations and License Suspensions

Rates of moving violations among drivers with mood disorders were greater than among those without mood disorders (48-month aRR, 1.25 [95% CI, 1.13-1.38]). GDL violations were similar between drivers with and without mood disorders (Figure 4; eTable 5 in Supplement 1). In the 48 months after licensure, 141 of 1343 drivers with mood disorders (10.5%) and 4729 of 69 595 drivers without mood disorders (6.8%) had their license suspended at least once (*P* < .001); mean suspension rates were 12.8 and 5.7 days per driver-year, respectively (48-month aRR, 1.95 [95% CI, 1.53-2.49]).

## Discussion

To our knowledge, this cohort study is the first study to use a longitudinal cohort with objective outcome measures to report on licensure and driving outcomes for youths with mood disorders. Youths with mood disorders were less likely to obtain a license in the immediate years after licensure eligibility compared with their counterparts without mood disorders. Among those licensed, youths with mood disorders demonstrated increased rates of adverse driving outcomes, including rates of night crashes, moving violations, and license suspensions, compared with peers without mood disorders.

Initial licensure acquisition is an important life event for many youths, and avoiding delays in licensure attainment has been associated with higher self-reports of health and educational attainment in young adults.^39^ Our study highlights how youths with mood disorders are less likely to achieve this milestone. Given that strength of interpersonal relationships, mobility, and self-sufficiency are protective factors against depression,^1,2^ failure to acquire a license could be associated with adverse psychosocial outcomes for youths with mood disorders.

In older adults, driving cessation has been associated with higher self-reported levels of depression.^40^ Depressive symptoms in this population were associated with a decreased sense of self-control owing to reduced mobility.^41,42^ Longitudinal studies suggest that depression typically follows rather than precedes driving cessation in this population,^40,43^ although a bidirectional association may exist.^41,42,44,45^ Few studies have attempted to examine driving avoidance and mood disorders in youths and thus far have not identified an association.^44^ Symptoms of depression, such as anhedonia and attention difficulties, can make the steps to acquire a license challenging. Further work is needed to clarify underlying mechanisms associated with decreased rates of initial license acquisition in adolescents and young adults with mood disorders.

We report an increase in risk for experiencing certain types of crashes among youths with mood disorders, as well as higher rates of driving outcomes that are associated with risky driving behaviors (eg, moving violations). Mood disorders may be associated with increased driver error via impaired concentration, reduced decisiveness, or changes in motivation.^2,15^ Alternatively, strong emotional responses and impaired regulation, which are often components of mood disorders, have been associated with risky driving behaviors.^46,47,48^ However, risk of other driving outcomes, including GDL citations and peer-passenger crashes, did not differ between our study groups. Interactions among mental health, risk-taking, and the driving context are incompletely elucidated and are a critical area of ongoing study.^46,48^

Pharmacotherapy is commonly used in the treatment of mood disorders, and multiple classes of medications exist. Prior studies evaluating the association of medications with crash risk among patients with ADHD have yielded mixed results.^49,50^ The role of medications and their association with driving outcomes in youths with mood disorders warrants further investigation.

Our study highlights the value of developing tailored guidance for families of youths with mood disorders. Given that driving can provide access to psychosocial benefits, licensure can be important for youths. Currently, most family-directed driving interventions are formulated for the general population.^51^ Clinicians taking care of youths with mood disorders should discuss current best practices on driving and licensure outcomes with their patients and families. Shared decision-making conversations may help families determine the best balance of autonomy and safety among youths of driving age. Given the delay in license acquisition, with a slight increase in hazardous driving behavior, opportunities may exist to enhance driving safety in this population.

### Limitations

There are several limitations to our study. Mood disorder diagnoses relied on clinician assessment rather than more rigorous criteria in the *Diagnostic and Statistical Manual of Mental Disorders* (*Fifth Edition)*.^52^ Additionally, mood disorders are fluid conditions, with periods of active episodes, remission, and recurrence^1,2^; some individuals in the mood disorder cohort may not have had mood disorder–related impairments at or after licensure. To mitigate this, we limited our cohort to individuals with mood disorder diagnoses in the 2 years (ie, ages 15-16 years) immediately preceding licensure eligibility to maximize capture of current symptoms. Conversely, some individuals in our no mood disorder cohort may have developed mood disorders during the follow-up period. Outcomes associated with mood disorder severity, pharmacotherapy, or co-occurring substance use or mental health conditions beyond anxiety and ADHD in our cohort were not assessed and are an opportunity for future study. Additionally, some youths may not have been diagnosed with mood disorders given that there are disparities in identification of particular depressive symptoms and disorders.^53^ Improving our understanding of low-incident psychiatric disorders, such as bipolar disorders, and driving outcomes is an area of future study.

Traffic crashes that did not result in bodily injury or damage in excess of $500 or those that were not reported to the police were not captured by our study. Driving exposure was not directly measured, although it is an important variable given rapid declines in crash rates in the initial years after licensure. Our analyses, however, accounted for time since licensure, which has been used as a proxy for exposure. Adolescents and young adults may move out of state after high school and thus could not be followed. Evidence describing differences in educational attainment between youths with and without mood disorders is mixed.^54,55^ In our study, associations were constant over time and included the immediate postlicensure period, when most individuals were still in high school. Given that New Jersey has the oldest licensing age in the US, as well as high levels of urbanization, the generalizability of our findings may be limited.

## Conclusions

This cohort study found that youths with mood disorders were less likely to be licensed and experienced higher rates of other adverse driving outcomes. Addressing social isolation and encouraging self-sufficiency is critical for youths with mood disorders, and driving may facilitate these aims. Fostering autonomy concurrently with safe mobility is an important and sensitive challenge in this population. To build on this foundational study, further research is needed to elucidate underlying mechanisms by which mood disorders are associated with licensure and driving outcomes, as well as the association of pharmacotherapy with modified outcomes, to best develop targeted interventions.

## Notes

Supplement 1. **eTable 1.** List of *ICD-9-CM* and *ICD-10-CM* Codes Used to Define Study Cohort **eTable 2.** Demographic Characteristics of Study Cohort by Mood Disorder Status and Sex **eTable 3.** Unadjusted Cumulative Probability of Licensure and First Crash Involvement by Mood Disorder Status **eTable 4.** Unadjusted Cumulative Probability of Licensure and First Crash Involvement by Mood Disorder Status and Sex **eTable 5.** Mean Monthly Rates, Crude Rate Ratios, and Adjusted Ratio Ratios for Crash and Violation Outcomes Comparing Drivers With and Without Mood Disorders

Supplement 2.
Data Sharing Statement
