# Supplementary material for: Driver’s Licensure and Driving Outcomes Among Youths With Mood Disorders
Source: JAMA Netw Open. 2024 Apr 8;7(4):e245543. doi: 10.1001/jamanetworkopen.2024.5543 (PMC11002704; doi:10.1001/jamanetworkopen.2024.5543)
Supplement: Supplement 1. — eTable 1. List of ICD-9-CM and ICD-10-CM Codes Used to Define Study Cohort eTable 2. Demographic Characteristics of Study Cohort by Mood Disorder Status and Sex eTable 3. Unadjusted Cumulative Probability of Licensure and First Crash Involvement by Mood Disorder Status eTable 4. Unadjusted Cumulative Probability of Licensure and First Crash Involvement by Mood Disorder Status and Sex eTable 5. Mean Monthly Rates, Crude Rate Ratios, and Adjusted Ratio Ratios for Crash and Violation Outcomes Comparing Drivers With and Without Mood Disorders [file jamanetwopen-e245543-s001.pdf]

# Supplemental Online Content

Gaw CE, Metzger KB, Pfeiffer MR, et al. Driver's licensure and driving outcomes among youths with mood disorders. *JAMA Netw Open*. 2024;7(4):e245543. doi:10.1001/jamanetworkopen.2024.5543

**eTable 1.** List of *ICD-9-CM* and *ICD-10-CM* Codes Used to Define Study Cohort

**eTable 2.** Demographic Characteristics of Study Cohort by Mood Disorder Status and Sex

**eTable 3.** Unadjusted Cumulative Probability of Licensure and First Crash Involvement by Mood Disorder Status

**eTable 4.** Unadjusted Cumulative Probability of Licensure and First Crash Involvement by Mood Disorder Status and Sex

**eTable 5.** Mean Monthly Rates, Crude Rate Ratios, and Adjusted Ratio Ratios for Crash and Violation Outcomes Comparing Drivers With and Without Mood Disorders

This supplemental material has been provided by the authors to give readers additional information about their work.

© 2024 Gaw CE et al. *JAMA Network Open.*

**eTable 1.** List of ICD-9-CM and ICD-10-CM Codes Used to Define Study Cohort

*Abbreviations*: ICD-9-CM— International Classification of Diseases, Ninth Revision, Clinical Modification; ICD-10-CM—International Statistical Classification of Diseases, Tenth Revision, Clinical Modification.

Intellectual Disability ICD-9-CM and ICD-10-CM Codes

| **Code** | **Condition** | **Source for code: ICD-9-CM or ICD-10-CM** |
|---|---|---|
| 3158 | Other specified delays in development | 9 |
| 3159 | Unspecified delay in development | 9 |
| 317 | Mild intellectual disabilities | 9 |
| 3180 | Moderate intellectual disabilities | 9 |
| 3181 | Severe intellectual disabilities | 9 |
| 3182 | Profound intellectual disabilities | 9 |
| 319 | Unspecified intellectual disabilities | 9 |
| F70 | Mild intellectual disabilities | 10 |
| F71 | Moderate intellectual disabilities | 10 |
| F72 | Severe intellectual disabilities | 10 |
| F73 | Profound intellectual disabilities | 10 |
| F78 | Other intellectual disabilities | 10 |
| F79 | Unspecified intellectual disabilities | 10 |
| F819 | Developmental disorder of scholastic skills, unspecified | 10 |
| F88 | Other disorders of psychological development | 10 |
| F89 | Unspecified disorder of psychological development | 10 |

* ICD-9-CM and ICD-10-CM codes were bridged using General Equivalence Mappings provided by the US Centers for Medicare and Medicaid Services.

Mood Disorder ICD-9-CM and ICD-10-CM Codes

| **Code** | **Condition** | **Source for code: ICD-9CM or ICD-10CM** |
|---|---|---|
| 29600 | Bipolar I disorder, single manic episode, unspecified | 9 |
| 29601 | Bipolar I disorder, single manic episode, mild | 9 |
| 29602 | Bipolar I disorder, single manic episode, moderate | 9 |

© 2024 Gaw CE et al. *JAMA Network Open.*

| | | |
|---|---|---|
| 29603 | Bipolar I disorder, single manic episode, severe, without mention of psychotic behavior | 9 |
| 29604 | Bipolar I disorder, single manic episode, severe, specified as with psychotic behavior | 9 |
| 29605 | Bipolar I disorder, single manic episode, in partial or unspecified remission | 9 |
| 29610 | Manic affective disorder, recurrent episode, unspecified | 9 |
| 29611 | Manic affective disorder, recurrent episode, mild | 9 |
| 29612 | Manic affective disorder, recurrent episode, moderate | 9 |
| 29613 | Manic affective disorder, recurrent episode, severe, without mention of psychotic behavior | 9 |
| 29614 | Manic affective disorder, recurrent episode, severe, specified as with psychotic behavior | 9 |
| 29615 | Manic affective disorder, recurrent episode, in partial or unspecified remission | 9 |
| 29620 | Major depressive affective disorder, single episode, unspecified | 9 |
| 29621 | Major depressive affective disorder, single episode, mild | 9 |
| 29622 | Major depressive affective disorder, single episode, moderate | 9 |
| 29623 | Major depressive affective disorder, single episode, severe, without mention of psychotic behavior | 9 |
| 29624 | Major depressive affective disorder, single episode, severe, specified as with psychotic behavior | 9 |
| 29625 | Major depressive affective disorder, single episode, in partial or unspecified remission | 9 |
| 29630 | Major depressive affective disorder, recurrent episode, unspecified | 9 |
| 29631 | Major depressive affective disorder, recurrent episode, mild | 9 |
| 29632 | Major depressive affective disorder, recurrent episode, moderate | 9 |
| 29633 | Major depressive affective disorder, recurrent episode, severe, without mention of psychotic behavior | 9 |
| 29634 | Major depressive affective disorder, recurrent episode, severe, specified as with psychotic behavior | 9 |
| 29635 | Major depressive affective disorder, recurrent episode, in partial or unspecified remission | 9 |
| 29640 | Bipolar I disorder, most recent episode (or current) manic, unspecified | 9 |
| 29641 | Bipolar I disorder, most recent episode (or current) manic, mild | 9 |
| 29642 | Bipolar I disorder, most recent episode (or current) manic, moderate | 9 |
| 29643 | Bipolar I disorder, most recent episode (or current) manic, severe, without mention of psychotic behavior | 9 |
| 29644 | Bipolar I disorder, most recent episode (or current) manic, severe, specified as with psychotic behavior | 9 |
| 29645 | Bipolar I disorder, most recent episode (or current) manic, in partial or unspecified remission | 9 |

© 2024 Gaw CE et al. *JAMA Network Open.*

| | | |
|---|---|---|
| 29650 | Bipolar I disorder, most recent episode (or current) depressed, unspecified | 9 |
| 29651 | Bipolar I disorder, most recent episode (or current) depressed, mild | 9 |
| 29652 | Bipolar I disorder, most recent episode (or current) depressed, moderate | 9 |
| 29653 | Bipolar I disorder, most recent episode (or current) depressed, severe, without mention of psychotic behavior | 9 |
| 29654 | Bipolar I disorder, most recent episode (or current) depressed, severe, specified as with psychotic behavior | 9 |
| 29655 | Bipolar I disorder, most recent episode (or current) depressed, in partial or unspecified remission | 9 |
| 29660 | Bipolar I disorder, most recent episode (or current) mixed, unspecified | 9 |
| 29661 | Bipolar I disorder, most recent episode (or current) mixed, mild | 9 |
| 29662 | Bipolar I disorder, most recent episode (or current) mixed, moderate | 9 |
| 29663 | Bipolar I disorder, most recent episode (or current) mixed, severe, without mention of psychotic behavior | 9 |
| 29664 | Bipolar I disorder, most recent episode (or current) mixed, severe, specified as with psychotic behavior | 9 |
| 29665 | Bipolar I disorder, most recent episode (or current) mixed, in partial or unspecified remission | 9 |
| 2967 | Bipolar I disorder, most recent episode (or current) unspecified | 9 |
| 29680 | Bipolar disorder, unspecified | 9 |
| 29681 | Atypical manic disorder | 9 |
| 29682 | Atypical depressive disorder | 9 |
| 29689 | Other bipolar disorders | 9 |
| 29690 | Unspecified episodic mood disorder | 9 |
| 29699 | Other specified episodic mood disorder | 9 |
| 3004 | Dysthymic disorder | 9 |
| 311 | Depressive disorder, not elsewhere classified | 9 |
| 6254 | Premenstrual tension syndromes | 9 |
| F3010 | Manic episode without psychotic symptoms, unspecified | 10 |
| F3011 | Manic episode without psychotic symptoms, mild | 10 |
| F3012 | Manic episode without psychotic symptoms, moderate | 10 |
| F3013 | Manic episode, severe, without psychotic symptoms | 10 |
| F302 | Manic episode, severe with psychotic symptoms | 10 |

© 2024 Gaw CE et al. *JAMA Network Open.*

| | | |
|---|---|---|
| F303 | Manic episode in partial remission | 10 |
| F308 | Other manic episodes | 10 |
| F309 | Manic episode, unspecified | 10 |
| F310 | Bipolar disorder, current episode hypomanic | 10 |
| F3110 | Bipolar disorder, current episode manic without psychotic features, unspecified | 10 |
| F3111 | Bipolar disorder, current episode manic without psychotic features, mild | 10 |
| F3112 | Bipolar disorder, current episode manic without psychotic features, moderate | 10 |
| F3113 | Bipolar disorder, current episode manic without psychotic features, severe | 10 |
| F312 | Bipolar disorder, current episode manic severe with psychotic features | 10 |
| F3130 | Bipolar disorder, current episode depressed, mild or moderate severity, unspecified | 10 |
| F3131 | Bipolar disorder, current episode depressed, mild | 10 |
| F3132 | Bipolar disorder, current episode depressed, moderate | 10 |
| F314 | Bipolar disorder, current episode depressed, severe, without psychotic features | 10 |
| F315 | Bipolar disorder, current episode depressed, severe, with psychotic features | 10 |
| F3160 | Bipolar disorder, current episode mixed, unspecified | 10 |
| F3161 | Bipolar disorder, current episode mixed, mild | 10 |
| F3162 | Bipolar disorder, current episode mixed, moderate | 10 |
| F3163 | Bipolar disorder, current episode mixed, severe, without psychotic features | 10 |
| F3164 | Bipolar disorder, current episode mixed, severe, with psychotic features | 10 |
| F3171 | Bipolar disorder, in partial remission, most recent episode hypomanic | 10 |
| F3173 | Bipolar disorder, in partial remission, most recent episode manic | 10 |
| F3175 | Bipolar disorder, in partial remission, most recent episode depressed | 10 |
| F3177 | Bipolar disorder, in partial remission, most recent episode mixed | 10 |
| F3181 | Bipolar II disorder | 10 |
| F3189 | Other bipolar disorder | 10 |
| F319 | Bipolar disorder, unspecified | 10 |
| F320 | Major depressive disorder, single episode, mild | 10 |
| F321 | Major depressive disorder, single episode, moderate | 10 |
| F322 | Major depressive disorder, single episode, severe without psychotic features | 10 |
| F323 | Major depressive disorder, single episode, severe with psychotic features | 10 |

© 2024 Gaw CE et al. *JAMA Network Open.*

| | | |
|---|---|---|
| F324 | Major depressive disorder, single episode, in partial remission | 10 |
| F3281 | Premenstrual dysphoric disorder | 10 |
| F3289 | Other specified depressive episodes | 10 |
| F329 | Major depressive disorder, single episode, unspecified | 10 |
| F330 | Major depressive disorder, recurrent, mild | 10 |
| F331 | Major depressive disorder, recurrent, moderate | 10 |
| F332 | Major depressive disorder, recurrent severe without psychotic features | 10 |
| F333 | Major depressive disorder, recurrent, severe with psychotic symptoms | 10 |
| F3341 | Major depressive disorder, recurrent, in partial remission | 10 |
| F338 | Other recurrent depressive disorders | 10 |
| F339 | Major depressive disorder, recurrent, unspecified | 10 |
| F341 | Dysthymic disorder | 10 |
| F348 | Other persistent mood [affective] disorders | 10 |
| F349 | Persistent mood [affective] disorder, unspecified | 10 |
| F39 | Unspecified mood [affective] disorder | 10 |
| N943 | Premenstrual tension syndrome | 10 |

* ICD-9-CM and ICD-10-CM codes were bridged using General Equivalence Mappings provided by the US Centers for Medicare and Medicaid Services.

© 2024 Gaw CE et al. *JAMA Network Open.*

**eTable 2.** Demographic Characteristics of Study Cohort by Mood Disorder Status and Sex

| | | Females | | | Males | | |
|---|---|---|---|---|---|---|---|
| | Overall Study Population N=86,173 | Mood Disorder N=1,226 | No Mood Disorder N=41,668 | P value | Mood Disorder N=653 | No Mood Disorder N=42,623 | P value |
| Age at last visit before age 17, median (IQR), y | 15.0 (14.0-16.0) | 16.0 (16.0-16.0) | 15.0 (14.0-16.0) | <0.001 | 16.0 (16.0-16.0) | 15.0 (14.0-16.0) | <0.001 |
| Age at end of study period, median (IQR), y | 22.8 (19.7-26.5) | 20.9 (18.7-24.4) | 22.8 (19.7-26.5) | <0.001 | 21.8 (19.3-25.5) | 22.9 (19.8-26.6) | <0.001 |
| Race/ethnicity, n (%) | | | | <0.001 | | | 0.002 |
| Hispanic | 8,629 (10.0) | 157 (12.8) | 4,162 (10.0) | | 78 (11.9) | 4,232 (9.9) | |
| Non-Hispanic Black | 8,852 (10.3) | 78 (6.4) | 4,135 (9.9) | | 48 (7.4) | 4,591 (10.8) | |
| Non-Hispanic White | 61,549 (71.4) | 894 (72.9) | 29,853 (71.6) | | 487 (74.6) | 30,315 (71.1) | |
| Non-Hispanic Other [a] | 7,113 (8.3) | 97 (7.9) | 3,496 (8.4) | | 40 (6.1) | 3,479 (8.2) | |
| Unknown | 30 (<0.1) | 0 | 22 (0.1) | | 0 | 6 (<0.1) | |
| Payor at last visit, n (%) | | | | 0.01 | | | <0.001 |
| Private | 74,408 (86.3) | 1097 (89.5) | 36,186 (86.8) | | 544 (83.3) | 36,578 (85.8) | |
| Medicaid | 5,943 (6.9) | 105 (8.6) | 2,647 (6.4) | | 74 (11.3) | 3,117 (7.3) | |
| Self-pay, not recorded, or not billed | 5,822 (6.8) | 24 (2.0) | 2,835 (6.8) | | 35 (5.4) | 2,928 (6.9) | |
| Median household income of residential census tract, $, n (%) | | | | 0.20 | | | 0.39 |
| < $48,506 | 6,633 (7.7) | 89 (7.3) | 3,064 (7.4) | | 51 (7.8) | 3,426 (8.0) | |
| $48,506 - $66,937 | 15,319 (17.8) | 200 (16.3) | 7,336 (17.6) | | 101 (15.5) | 7,682 (18.0) | |
| $66,938 - $84,921 | 22,324 (25.9) | 348 (28.4) | 10,818 (26.0) | | 177 (27.1) | 10,981 (25.8) | |
| $84,922 - $110,035 | 23,510 (27.3) | 348 (28.4) | 11,491 (27.6) | | 190 (29.1) | 11,481 (26.9) | |
| ≥ $110,036 | 18,377 (21.3) | 241 (19.7) | 8,956 (21.5) | | 134 (20.5) | 9,046 (21.2) | |
| Unknown | 10 (<0.1) | 0 | 3 (<0.1) | | 0 | 7 (<0.1) | |
| Neighborhood population density, population per square mile, n (%) | | | | 0.42 | | | 0.46 |
| < 1,299 | 29,994 (34.8) | 436 (35.6) | 14,637 (35.1) | | 224 (34.3) | 14697 (34.5) | |
| 1,299 - 2,919 | 27,439 (31.8) | 405 (33.0) | 13,331 (32.0) | | 218 (33.4) | 13485 (31.6) | |
| 2,920 - 5,237 | 19,999 (23.2) | 260 (21.2) | 9,523 (22.9) | | 155 (23.7) | 10061 (23.6) | |
| 5,238 - 12,711 | 7,537 (8.7) | 114 (9.3) | 3,630 (8.7) | | 51 (7.8) | 3739 (8.8) | |
| ≥ 12,712 | 1,202 (1.4) | 11 (0.9) | 546 (1.3) | | 5 (0.8) | 640 (1.5) | |
| Unknown | 2 (<0.1) | 0 | 1 (<0.1) | | 0 | 1 (<0.1) | |
| Co-occurring anxiety disorder at age 15-16, n (%) | | | | <0.001 | | | <0.001 |
| No | 84,406 (97.9) | 813 (66.3) | 40,938 (98.2) | | 493 (75.5) | 42,159 (98.9) | |
| Yes | 1,767 (2.1) | 413 (33.7) | 730 (1.8) | | 160 (24.5) | 464 (1.1) | |
| Co-occurring attention deficit hyperactivity disorder, n (%) | | | | <0.001 | | | <0.001 |
| No | 78,897 (91.6) | 978 (79.8) | 39,803 (95.5) | | 389 (59.6) | 37,724 (88.5) | |
| Yes | 7,276 (8.4) | 248 (20.2) | 1,865 (4.5) | | 264 (40.4) | 4,899 (11.5) | |

[a] Non-Hispanic other includes individuals who were categorized as either not Hispanic and 1 race (American Indian or Alaska Native, Asian or Indian, Native Hawaiian or Other Pacific Islander, or other) or non Hispanic and 2 or more of any race.

© 2024 Gaw CE et al. *JAMA Network Open.*

**eTable 3.** Unadjusted Cumulative Probability of Licensure and First Crash Involvement by Mood Disorder Status

| | Mood Disorder Status | | | |
|---|---|---|---|---|
| | Mood Disorder | | No Mood Disorder | |
| **Outcome** | No. [a] | Cumulative Probability (95% CI) [b] | No. [a] | Cumulative Probability (95% CI) [b] |
| License acquired post-eligibility by | | | | |
| Number at beginning of follow up | (n=1,879) | | (n=84,294) | |
| 1 mo | 570 | 0.30 (0.28, 0.32) | 39,591 | 0.47 (0.47, 0.47) |
| 12 mo | 1,062 | 0.58 (0.56, 0.60) | 60,511 | 0.73 (0.72, 0.73) |
| 24 mo | 1,224 | 0.69 (0.66, 0.71) | 65,982 | 0.80 (0.80, 0.80) |
| 36 mo | 1,279 | 0.73 (0.71, 0.75) | 67,645 | 0.83 (0.82, 0.83) |
| 48 mo | 1,304 | 0.76 (0.73, 0.78) | 68,409 | 0.84 (0.84, 0.84) |
| First crash involvement post-licensure by | | | | |
| Number at beginning of follow up | (n=1,343) | | (n=69,595) | |
| 12 mo | 227 | 0.18 (0.16, 0.21) | 10,823 | 0.16 (0.16, 0.17) |
| 24 mo | 331 | 0.28 (0.26, 0.31) | 15,955 | 0.25 (0.25, 0.25) |
| 36 mo | 382 | 0.34 (0.31, 0.37) | 19,070 | 0.31 (0.31, 0.31) |
| 48 mo | 428 | 0.41 (0.38, 0.44) | 21,138 | 0.36 (0.35, 0.36) |

[a] Indicates the number of individuals who achieved the outcome at a specific post-eligibility or post-licensure month.
[b] Kaplan-Meier survival curves were used to estimate the cumulative probability of licensure and crash over time.

© 2024 Gaw CE et al. *JAMA Network Open.*

**eTable 4**. Unadjusted Cumulative Probability of Licensure and First Crash Involvement by Mood Disorder Status and Sex

| | Females | | | | Males | | | |
|---|---|---|---|---|---|---|---|---|
| | Mood Disorder | | No Mood Disorder | | Mood Disorder | | No Mood Disorder | |
| **Outcome** | No. [a] | Cumulative Probability (95% CI) [b] | No. [a] | Cumulative Probability (95% CI) [b] | No. [a] | Cumulative Probability (95% CI) [b] | No. [a] | Cumulative Probability (95% CI) [b] |
| License acquired post-eligibility by | | | | | | | | |
| Number at beginning of follow up | (n=1,226) | | (n=41,668) | | (n=653) | | (n=42,623) | |
| 1 mo | 418 | 0.34 (0.32, 0.37) | 20,980 | 0.50 (0.50, 0.51) | 152 | 0.23 (0.20, 0.27) | 18,611 | 0.44 (0.43, 0.44) |
| 12 mo | 728 | 0.61 (0.58, 0.64) | 30,939 | 0.75 (0.75, 0.76) | 334 | 0.53 (0.49, 0.57) | 29,572 | 0.70 (0.70, 0.71) |
| 24 mo | 839 | 0.72 (0.70, 0.75) | 33,437 | 0.82 (0.82, 0.82) | 385 | 0.62 (0.58, 0.66) | 32,545 | 0.78 (0.78, 0.79) |
| 36 mo | 867 | 0.76 (0.73, 0.79) | 34,186 | 0.84 (0.84, 0.85) | 409 | 0.67 (0.63, 0.71) | 33,459 | 0.81 (0.81, 0.81) |
| 48 mo | 882 | 0.78 (0.76, 0.81) | 34,558 | 0.85 (0.85, 0.86) | 422 | 0.70 (0.66, 0.74) | 33,851 | 0.82 (0.82, 0.83) |
| First crash involvement post-licensure by | | | | | | | | |
| Number at beginning of follow up | (n=903) | | (n=35,134) | | (n=440) | | (n=34,461) | |
| 12 mo | 146 | 0.18 (0.15, 0.20) | 5,417 | 0.16 (0.16, 0.17) | 81 | 0.20 (0.16, 0.24) | 5,406 | 0.16 (0.16, 0.17) |
| 24 mo | 212 | 0.27 (0.24, 0.31) | 7,941 | 0.25 (0.24, 0.25) | 119 | 0.30 (0.26, 0.35) | 8,014 | 0.25 (0.25, 0.26) |
| 36 mo | 246 | 0.33 (0.30, 0.37) | 9,489 | 0.31 (0.30, 0.31) | 136 | 0.36 (0.31, 0.41) | 9,581 | 0.32 (0.31, 0.32) |
| 48 mo | 277 | 0.40 (0.36, 0.44) | 10,490 | 0.35 (0.34, 0.36) | 151 | 0.42 (0.37, 0.47) | 10,648 | 0.36 (0.36, 0.37) |

[a] Indicates the number of individuals who achieved the outcome at a specific post-eligibility or post-licensure month.
[b] Kaplan-Meier survival curves were used to estimate the cumulative probability of licensure and crash over time.

© 2024 Gaw CE et al. *JAMA Network Open.*

**eTable 5.** Average Monthly Rates, Crude Rate Ratios, and Adjusted Ratio Ratios for Crash and Violation Outcomes Comparing Drivers with and without Mood Disorders

| | | Months of licensure | | | | | | | | | | | |
|---|---|---|---|---|---|---|---|---|---|---|---|---|---|
| | | 12 | | | | | | 48 | | | | | |
| **Among All Drivers** | | **Mood Disorder** | | **No Mood Disorder** | | | | **Mood Disorder** | | **No Mood Disorder** | | | |
| | Event type | Number of events | Crude rate per 10,000 driver-months | Number of events | Crude rate per 10,000 driver-months | cRR (95% CI) | aRR (95% CI) [a] | Number of events | Crude rate per 10,000 driver-months | Number of events | Crude rate per 10,000 driver-months | cRR (95% CI) | aRR (95% CI) [a] |
| Crash | All crashes | 271 | 185.3 | 11,895 | 150.4 | 1.23 (1.08, 1.40) | 1.16 (1.01, 1.34) | 618 | 137.8 | 27,681 | 104.8 | 1.31 (1.20, 1.44) | 1.19 (1.08, 1.31) |
| | At-fault crashes | 206 | 140.9 | 8,516 | 107.6 | 1.31 (1.13, 1.52) | 1.23 (1.05, 1.44) | 449 | 100.2 | 18,514 | 70.1 | 1.43 (1.29, 1.59) | 1.30 (1.16, 1.45) |
| | Peer-passenger crashes | 64 | 43.8 | 3,658 | 46.2 | 0.95 (0.74, 1.21) | 0.98 (0.75, 1.28) | 103 | 23.0 | 6,241 | 23.6 | 0.97 (0.80, 1.18) | 0.99 (0.80, 1.22) |
| | Night crashes (9:01pm-4:59am) | 38 | 26.0 | 1,388 | 17.5 | 1.48 (1.05, 2.09) | 1.61 (1.12, 2.34) | 76 | 17.0 | 3,558 | 13.5 | 1.26 (0.99, 1.60) | 1.28 (0.99, 1.67) |
| | Injury crashes | 10 | 6.8 | 590 | 7.5 | 0.92 (0.49, 1.71) | 0.77 (0.40, 1.51) | 34 | 7.6 | 1,445 | 5.5 | 1.39 (0.98, 1.96) | 1.29 (0.89, 1.87) |
| | Alcohol-related crashes | 4 | 2.7 | 65 | 0.8 | 3.33 (1.21, 9.13) | 3.54 (1.27, 9.86) [b] | 15 | 3.3 | 263 | 1.0 | 3.36 (2.00, 5.64) | 3.66 (2.18, 6.14) [b] |
| Citation | Moving violation citations | 330 | 225.7 | 13,856 | 175.1 | 1.29 (1.12, 1.48) | 1.31 (1.13, 1.52) | 959 | 213.9 | 44,320 | 167.9 | 1.27 (1.16, 1.40) | 1.25 (1.13, 1.38) |
| | GDL citations | 32 | 22.5 | 1,951 | 25.1 | 0.90 (0.62, 1.31) | 1.07 (0.73, 1.56) [b] | - | - | - | - | - | - |
| Suspension | Suspensions [c] | 3,364 | 2.7 | 79,622 | 1.2 | 2.28 (1.47, 3.53) | 2.18 (1.35, 3.54) | 49,479 | 12.8 | 1,263,822 | 5.7 | 2.26 (1.81, 2.82) | 1.95 (1.53, 2.49) |
| **Among Female Drivers** | | | | | | | | | | | | | |
| Crash | All crashes | 165 | 167.8 | 5,930 | 148.2 | 1.13 (0.96, 1.33) | 1.06 (0.89, 1.26) | 390 | 131.6 | 13,567 | 101.3 | 1.30 (1.16, 1.46) | 1.16 (1.02, 1.32) |
| | At-fault crashes | 125 | 127.1 | 4,138 | 103.4 | 1.23 (1.03, 1.47) | 1.13 (0.93, 1.39) | 278 | 93.8 | 8,772 | 65.5 | 1.43 (1.25, 1.64) | 1.26 (1.09, 1.45) |

© 2024 Gaw CE et al. *JAMA Network Open.*

| | | | | | | | | | | | | | |
|---|---|---|---|---|---|---|---|---|---|---|---|---|---|
| | Peer-passenger crashes | 38 | 38.6 | 1,757 | 43.9 | 0.88 (0.64, 1.21) | 0.92 (0.65, 1.31) | 60 | 20.2 | 2,878 | 21.5 | 0.94 (0.73, 1.22) | 0.94 (0.72, 1.24) |
| | Night crashes (9:01pm-4:59am) | 16 | 16.3 | 623 | 15.6 | 1.04 (0.62, 1.76) | 1.09 (0.61, 1.97) | 41 | 13.8 | 1,583 | 11.8 | 1.17 (0.84, 1.63) | 1.14 (0.79, 1.64) |
| | Injury crashes | 8 | 8.1 | 288 | 7.2 | 1.13 (0.56, 2.27) | 1.11 (0.55, 2.24) [b] | 20 | 6.7 | 661 | 4.9 | 1.37 (0.86, 2.17) | 1.23 (0.74, 2.03) |
| | Alcohol-related crashes | 1 | 1.0 | 15 | 0.4 | 2.71 (0.36, 20.5) | 2.64 (0.33, 21.0) [b] | 8 | 2.7 | 71 | 0.5 | 5.09 (2.45, 10.6) | 4.45 (2.13, 9.29) [b] |
| Citation | Moving violation citations | 175 | 177.9 | 5,072 | 126.8 | 1.40 (1.18, 1.67) | 1.32 (1.08, 1.60) | 506 | 170.7 | 16,740 | 125.0 | 1.37 (1.21, 1.54) | 1.26 (1.11, 1.44) |
| | GDL citations | 18 | 18.7 | 589 | 15 | 1.25 (0.79, 1.99) | 1.26 (0.79, 2.00) [b] | - | - | - | - | - | - |
| Suspension | Suspensions [c] | 1,437 | 1.7 | 20,102 | 0.6 | 2.90 (1.47, 5.72) | 1.95 (1.53, 2.49) | 23,654 | 9.3 | 424,820 | 3.8 | 2.48 (1.81, 3.40) | 2.04 (1.43, 2.90) |
| **Among Male Drivers** | | | | | | | | | | | | | |
| Crash | All crashes | 106 | 221.4 | 5,965 | 152.5 | 1.45 (1.16, 1.81) | 1.37 (1.08, 1.74) | 228 | 150.1 | 14,114 | 108.5 | 1.38 (1.19, 1.61) | 1.24 (1.06, 1.46) |
| | At-fault crashes | 81 | 169.2 | 4,378 | 112.0 | 1.51 (1.17, 1.95) | 1.40 (1.07, 1.82) | 171 | 112.6 | 9,742 | 74.9 | 1.50 (1.26, 1.79) | 1.34 (1.12, 1.62) |
| | Peer-passenger crashes | 26 | 54.3 | 1,901 | 48.6 | 1.12 (0.76, 1.65) | 1.09 (0.72, 1.65) | 43 | 28.3 | 3,363 | 25.9 | 1.09 (0.81, 1.49) | 1.06 (0.76, 1.47) |
| | Night crashes (9:01pm-4:59am) | 22 | 46 | 765 | 19.6 | 2.35 (1.50, 3.68) | 2.43 (1.53, 3.86) | 35 | 23.0 | 1,975 | 15.2 | 1.52 (1.06, 2.17) | 1.51 (1.03, 2.21) |
| | Injury crashes | 2 | 4.2 | 302 | 7.7 | 0.54 (0.14, 2.17) | 0.26 (0.04, 1.92) | 14 | 9.2 | 784 | 6.0 | 1.53 (0.91, 2.57) | 1.38 (0.79, 2.40) |
| | Alcohol-related crashes | 3 | 6.3 | 50 | 1.3 | 4.90 (1.53, 15.7) | 4.07 (1.27, 13.1) [b] | 7 | 4.6 | 192 | 1.5 | 3.12 (1.47, 6.62) | 3.01 (1.43, 6.35) [b] |
| Citation | Moving violation citations | 155 | 323.8 | 8,784 | 224.6 | 1.44 (1.16, 1.79) | 1.30 (1.03, 1.63) | 453 | 298.2 | 27,580 | 212.0 | 1.41 (1.21, 1.63) | 1.23 (1.05, 1.43) |
| | GDL citations | 14 | 30.3 | 1,362 | 35.4 | 0.85 (0.46, 1.59) | 0.89 (0.48, 1.66) [b] | - | - | - | - | - | - |

© 2024 Gaw CE et al. *JAMA Network Open.*

| | | | | | | | | | | | | | |
|---|---|---|---|---|---|---|---|---|---|---|---|---|---|
| Suspension | Suspensions [c] | 1,927 | 4.8 | 59,520 | 1.8 | 2.62 (1.48, 4.65) | 2.00 (1.06, 3.80) | 25,825 | 19.3 | 839,002 | 7.6 | 2.55 (1.88, 3.46) | 1.83 (1.30, 2.58) |

[a] Adjusted model includes the following covariates: sex, age at licensure, race/ethnicity, payor, anxiety, attention deficit hyperactivity disorder, birth year, and census tract-level household income and population density (except where noted).

[b] Fully adjusted model did not converge. Results are for a limited adjusted model that only includes the following covariates: sex and age at licensure.

[c] Suspension counts are measured in days. Rates are calculated by number of days with a suspended license divided by number of years of follow-up.

© 2024 Gaw CE et al. *JAMA Network Open.*
