# Supplementary material for: Driver’s Licensure and Driving Outcomes Among Youths With Mood Disorders
Source: JAMA Netw Open. 2024 Apr 8;7(4):e245543. doi: 10.1001/jamanetworkopen.2024.5543 (PMC11002704; doi:10.1001/jamanetworkopen.2024.5543)
Supplement: Supplement 2. — Data Sharing Statement [file jamanetwopen-e245543-s002.pdf]

Data Sharing Statement

Gaw. Driver's Licensure and Driving Outcomes Among Youths With Mood Disorders. *JAMA Netw Open*. Published online April 8, 2024. doi:10.1001/jamanetworkopen.2024.5543

Data
Data available: Yes
Data types: Data dictionary, Other (please specify)
Additional Information: Aggregate level data
How to access data: https://injury.research.chop.edu/new-jersey-safety-and-health-outcomes-data-warehouse
When available: With publication

Supporting Documents
Document types: None

Additional Information
Who can access the data: As all NJ-SHO activities (A) are bound by legal agreements between CHOP and data owners that specify stringent data security measures and (B) have been reviewed and approved by the Institutional Review Boards at CHOP and the NJ Department of Health, data can be made available to researchers whose proposed use of data has been approved.
Types of analyses: Specified purposes that are in line with data security measures and data use agreements.
Mechanisms of data availability: After proposal approval and with signed data access agreement
